# Domestic violence and its relationship with quality of life in pregnant women during the outbreak of COVID-19 disease

**DOI:** 10.1186/s12884-021-03579-x

**Published:** 2021-01-28

**Authors:** Somayyeh Naghizadeh, Mojgan Mirghafourvand, Roghaye Mohammadirad

**Affiliations:** 1 PhD Student of Reproductive Health, Midwifery and Reproductive Department, School of Nursing and Midwifery, Tehran University of Medical Sciences, Tehran, Iran; 2 Department of midwifery, Social Determinants of Health Research Centre, Tabriz University of Medical Sciences, Islamic Republic of Iran, 513897977, South Shariatie, Tabriz, Iran; 3 Department of Midwifery, 29 Bahman Hospital, Tabriz, Iran

**Keywords:** COVID-19, Pandemic, Domestic violence, Quality of Life, Pregnant women

## Abstract

**Background:**

During the COVID-19 pandemic, pregnant women bear considerable physical and psychological stress because of their special conditions, which combined with other stress factors such as violence, makes their situation even more critical. This study aimed to investigate the prevalence of domestic violence and its relationship with quality of life in pregnant women during the COVID-19 pandemic.

**Methods:**

This cross-sectional study was performed with the participation of 250 pregnant women in the obstetrics clinic of 29-Bahman Hospital, Tabriz city. Using a three-part questionnaire consisting of the socio-demographic and obstetrics information, the domestic violence questionnaire developed by WHO, and the SF-12 quality of life questionnaire, the required information was collected. A general linear model was then used to determine the relationship between domestic violence and quality of life, while adjusting the socio-demographic and obstetrics information.

**Results:**

According to the data, more than one-third of pregnant women (35.2 %) had experienced domestic violence. The most common type of violence experienced was emotional violence (32.8 %), followed by sexual violence (12.4 %), and physical violence (4.8 %). The mean score of the physical health department of quality of life in the group of women exposed to violence (50.21) was lower compared to the unexposed group (53.45), though there was no significant difference between them (*P* = 0.25). However, the mean score of the mental health department of quality of life in women exposed to violence (46.27) was significantly lower compared to unexposed women (61.17) (*P* < 0.001). Based on the general linear model, the mean score for quality of life in the mental health dimension was significantly higher among unexposed women compared to those exposed to violence (β = 9.3, 95 %CI: 3.5 to 15.0, *P* = 0.002).

**Conclusions:**

The findings of this study indicate a high prevalence of domestic violence and its relationship with a low quality of life during the COVID-19 pandemic. Therefore, the findings signify the importance of screening pregnant women in terms of domestic violence in respective centers as well as the necessity of conducting proper interventions to address domestic violence to improve the quality of life in women.

## Background

The newly-emerging COVID-19 pandemic is a new and spreading infectious and respiratory disease affecting all countries [1–3]. Due to the high transmission rate of this virus, the WHO has classified it as a pandemic infectious disease [4]. The current pandemic situation of COVID-19 worldwide is severe and concerning [5]. Individuals above 60 and those with a compromised immune system are more at risk of being affected by this disease [6], and since during pregnancy the immune system is relatively suppressed, pregnant women are more vulnerable to viral infections and their complications. Therefore, the COVID-19 pandemic may cause serious complications in pregnant women [3, 7]. Although there is not enough information available about the affliction of pregnant women and the complications associated with this disease, considering the physiological changes in the immune system and its relative suppression, as well as cardiopulmonary changes during pregnancy, pregnant women are more at the risk of infectious diseases and respiratory viruses [7]. All said factors lead to anxiety and stress in pregnant women and their families [3, 8]. Meanwhile, with the increasing spread of the disease in different countries including Iran, for personal protection and preventing the infection in vulnerable individuals, long-term quarantine and distancing are recommended [9]. Nevertheless, social isolation and severed communication with others can lead to feelings of discomfort, anxiety, panic, anger, resentment, despair, and domestic violence [10].

The World Health Organization (WHO) defines domestic violence as any violent and gender-dependent behavior causing damage or possible physical, sexual, emotional damage, or suffering for women. Such behaviors can occur with threats, as well as absolute deprivation of liberty and freedoms, and may occur implicitly or explicitly [11]. Worldwide, 30 % of women experience physical or sexual violence by their partner throughout their life [12]. Pregnancy alone imposes considerable psychological and physical stress on a woman, and when accompanied by other stress factors such as violence, they can adversely affect the health of mother and child. All said complications can increase maternal and neonatal mortality [1, 2]. Exposure to domestic violence during pregnancy is associated with numerous adverse consequences including prenatal bleeding, trauma to the fetus, congenital infection, uterine infection, atraumatic rupture of the spleen and pneumothorax, abortion, stillbirth, and premature delivery [13]. The negative consequences of psychological health including depression, anxiety, posttraumatic stress disorder, suicide, delayed visits for pregnancy care, maternal malnutrition, as well as drug abuse and chronic alcohol consumption, are associated with domestic violence during pregnancy [14].

Domestic violence may increase during human crises including conflicts and natural disasters [15]. Staying at home limits familial support, thus increasing social and functional isolation. There is ample evidence that social isolation increases the risk of victimization. Bright et al. [16] also reported that domestic violence increases with social isolation and in times of crisis.

In the course of the COVID-19 pandemic, more than half of the world’s population is asked to remain at home to slow down the spread of this disease [17]. For women who are currently exposed to violence or are at risk of such abuse, remaining at home increases the risk of violence committed by the marital partner [18].

Despite the scarcity of data, media coverage and the reports by organizations monitoring violence against women indicate an alarming image of increasing violence committed by the marital partner during the COVID-19 pandemic [19, 20]. Taub studied the concurrent problem of COVID-19 and domestic violence, stating that during the COVID-19 quarantine period, the risk of women’s exposure to domestic violence is higher due to the prolonged period couples have to spend together and their inability to leave home. Also, during the COVID-19 pandemic, following the end of home quarantine, with the decline in the economy, followed by personal crises such as job loss or serious financial problems, domestic violence becomes far more common [21]. In England, a project which tracked violence against women stated that the mortality resulting from domestic abuse became twice as much between 23 March and 12 April 2020, in comparison with the average of the past 10 years [22]. In Rio de Janeiro, the Attorney General Office of the government reported a 50 % increase in domestic violence in the first week following the enaction of social distancing across Brazil, with most complaints related to violence against women. Similarly, in the Parana State in Brazil, a 15 % increase in domestic violence was reported by the military police in the first week of social distancing [23].

Studies on the quality of life in pregnant women exposed to domestic violence indicate that it increases both short- and long-term negative health consequences [24]. According to WHO’s definition, quality of life refers to a person’s perception about their living condition in terms of culture, the value system in which they live, goals, expectations, standards, and priorities. It is a subjective concept that cannot be perceived by others; as it is based on a person’s perception of different aspects of their life [25]. The issue of quality of life in pregnant women exposed to violence is very important. Recently, the body of literature related to violence against women during pregnancy is growing in both developed and developing countries [26]; however, studies on the relationship between domestic violence and women’s quality of life in pregnancy and post-pregnancy periods are still scarce. Meanwhile, violence against women during pregnancy is an additional threat for physical and psychological health of both the mother and fetus and eventually, a threat to public health. Thus, diagnosing and preventing violence against pregnant women—especially in critical and disastrous times—such as the COVID-19 pandemic, are absolutely essential to improve the general state of women and their reproductive health. Therefore, the present study attempted to determine the prevalence of domestic violence and its association with quality of life during the COVID-19 pandemic among Iranian pregnant women.

## Methods

### Type of study and participants

This is a cross-sectional study, conducted in May-August 2020 during the outbreak of COVID-19 disease on pregnant women visiting the obstetrics clinic of 29-Bahman Hospital in Tabriz, Iran.

The inclusion criteria were as follows: willingness to participate in the study, Iranian nationality, residence in Tabriz city, gestational age ≥ 12 weeks to exclude abortion cases, singleton pregnancy, absence of any medical, obstetrics, or underlying risk factor or diagnosed disease/disorder, and absence of stressful events over the past six months, such as the death of one of the family members or divorce. The exclusion criteria were the following: incompletely filling the questionnaire items, fetal death, major anomalies in the fetus, excessive bleeding, fetal developmental disorders such as IUGR and macrosomia, a blood pressure of 140/90 mmHg or higher at the time of visit, as well as medical or obstetrics disorders in the mother (pregnancy-induced hypertension, chronic hypertension, overt diabetes, and gestational diabetes).

To determine the sample size in this study, a ratio estimation formula was used. Based on the findings of Tavoli et al. [27] regarding the frequency of violence against pregnant women (*p* = 0.64, d = 10 % around p, and α = 0.05), an initial sample size of 221 was obtained. Afterward, assuming a 10 % attrition, the sample size was adjusted to 250.

### Sampling

After acquiring the ethics permit from the ethics committee of Tabriz University of Medical Sciences (IR.TBZMED.REC.1399.332) and presenting the introduction letter to the authorities of the 29-Bahman hospital in Tabriz, the sampling permission was received. This study used convenience sampling for which the author visited the obstetrics clinic of 29-Bahman Hospital in Tabriz. Subsequently, all pregnant women over 12 weeks of gestation who met the inclusion criteria were registered and completed the questionnaires. Before recruiting the participants, they were informed about the goals and method of study, their voluntary participation, confidentiality, privacy protection, and the participant’s right to quit the study at any stage of data collection. Further, a corresponding waiver form was received from the participants. Immediately after completion, the questionnaires were verified by the author. Whenever the participants had a problem understanding the questionnaire items, the author carefully explained it to them.

### Data Collection tools

The data collection tool in this study was a three-part questionnaire, including the socio-demographic and obstetrics information questionnaire, the domestic violence questionnaire developed by WHO, and the SF12 quality of life questionnaire.

The socio-demographic and obstetrics information questionnaire captured age, level of education, occupation of the respondent and her spouse, duration of the marriage, place of residence, economic status, number of pregnancies, number of living children, gestational age, the effect of COVID-19 disease on receiving pregnancy care, being diagnosed with COVID-19, COVID-19 in relatives or acquaintances, the prolonged staying at the home of the spouse during the COVID-19 outbreak, reduction of the spouse income during the disease outbreak, aggressive behavior of the spouse during COVID-19 outbreak, and the effect of COVID-19 disease on the relationship with the spouse, and their self-rated health.

WHO’s domestic violence questionnaire measures the violence committed by the spouse in three areas consisting of physical, sexual, and emotional aspects [28]. In this questionnaire, physical, sexual, and emotional violence are assessed by 10, five, and 11 items, respectively. The cases of different types of violence were acquired based on a five-level Likert scale. A woman would be considered exposed to violence if she gave at least one positive response to each of the items related to the questionnaire capturing physical, sexual, or emotional violence. The women choosing the option “never”, were those who had not experienced violence. In this study, the women experiencing violence once or twice fell into the group of “mild violence”, while the women mentioning 3–5 times of experiencing violence would be categorized as “moderate violence”, and eventually the women who were exposed to violence more than five times would be classified in the “severe violence” group. The validity of the questionnaire in Iran has been confirmed by different researchers. For example, Hajian et al. [29] reported Cronbach’s alpha for the three areas of physical, psychological, and sexual violence as 0.92, 0.89, and 0.88, respectively. In this study, the Cronbach’s alpha for the three areas of physical, psychological, and sexual violence was obtained as 0.70, 0.78, and 0.79, respectively.

The SF-12 quality of life questionnaire is a multipurpose self-report questionnaire which is often used to investigate the quality of life and health. It is the shorter variant of the 36-item quality of life questionnaire. The 12-item version of the quality of life was designed in 1996 by Ware et al., [30] which has 12 items capturing eight areas consisting of physical functioning, social functioning, physical role constraints, emotional role constraints, psychological health, joy, physical pain, and general health. Considering the low number of items, normally the total score for each person is used. The score of the subject in each of these areas varies between 0 and 100, with higher scores representing a better quality of life. The reliability and validity of this questionnaire have been proven in different studies. For the first time, Ware et al. [30] investigated the reliability and validity of this questionnaire and reported respective Cronbach’s alphas of 0.89 and 0.76 for physical health and mental health dimensions. Montazeri et al. investigated the reliability and validity of this scale in Iran. The reliability of the 12 items of physical and psychological elements was reported as 0.73 and 0.72, respectively [31]. In the present study, the Cronbach’s alpha for the physical health and mental health dimensions was obtained as 0.65 and 0.75, respectively.

### Statistical analysis

After data collection, the data were analyzed with SPSS 21. The normality of quantitative data was tested based on kurtosis and skewness, where all data had a normal distribution. The acceptable range of values was less than 3 for skewness and less than 10 for kurtosis [32]. In this study, skewness was in the range of -0.89 to 0.11 for domestic violence types, and − 0.30 to 0.91 for quality of life subscales; while kurtosis was in the range of -0.79 to 0.94 for domestic violence types, and − 1.92 to 0.78 for quality of life subscales. To report the frequency of domestic violence and quality of life, descriptive statistics including frequency (percentage), mean, and standard deviation were used. Socio-demographic and obstetrics characteristics’ relationship with the frequency of domestic violence was examined using chi-square and independent t-tests. A general linear model was used to compare the quality of life in both exposed and unexposed groups, as well as for bivariate analysis, independent t-test, and multivariate analysis. Initially, the relationships between each socio-demographic and obstetrics characteristic and the total score of mental and physical health departments of quality of life were examined using one-way analysis of variance and independent t-test. After that, the variables related to the total score of the mental health department of quality of life with a p < 0.05, alongside the variable of violence, were inserted into the adjusted general linear model.

## Results

The mean and standard deviation of the age of pregnant women were respectively 30.57 and 5.87. More than half of the participants (53.2 %) were high school graduates. Most women (94.4 %) were housewives. The mean and standard deviation of the age of spouses were respectively 35.39 and 5.76. Less than half of the participants’ spouses (42.8 %) had a high school education. Moreover, five (2 %) of the women had a history of infection with the COVID-19 disease, while infection with the disease in the family members or relatives was reported in 21 individuals (8.4 %). Nearly one-third of participants (30.4 %) considered the outbreak of COVID-19 disease as a cause of declined pregnancy care. The mean and standard deviation of the marital period were respectively 8.74 and 5.09. Further, most participants (91.2 %) were urban residents, and most of them (78.4 %) stated their household economic status as moderate (Table 1).

**Table 1:** The socio-demographic and obstetrics characteristics of the participants (*n*= 250)

Characteristic	Number (Percent)
**Age** (Year)	
15-25	53 (21.2)
26-35	142 (56.8)
36-45	55 (22)
**Education**	
Illiterate	1 (0.4)
Elementary	16 (6.4)
Secondary	58 (23.2)
High school diploma	133 (53.2)
University	42 (16.8)
**Job**	
Housewife	236 (94.4)
Working at home	9 (3.6)
Working outside the home	5 (2)
**Husband’s age**	
20-30	54 (21.6)
31-40	148 (59.2)
≥40	48 (19.2)
**Husband’s education**	
Illiterate	7 (2.8)
Elementary	34 (13.6)
Secondary	62 (24.8)
High school diploma	107 (42.8)
University	40 (16)
**Husband’s job**	
Unemployed	6 (2.4)
Laborer	104 (41.6)
Employee	18 (7.2)
Freelance	122 (48.8)
**Household economic status**^**a**^	
Very poor	5 (2)
Poor	48 (19.2)
Moderate	196 (78.4)
Rich	1 (0.4)
**Duration of marriage** (Year)	
≤5	75 (30)
6-10	92 (36.8)
≥10	83 (33.2)
**Gravida**	
1	69 (27.6)
2	97 (38.8)
≥3	83 (33.6)
**Alive children**	
0	78 (31.2)
1	117 (46.8)
2	51 (20.7)
≥3	4 (1.6)
**Self-rated health status**	
Very good	36 (14.4)
Good	108 (43.2)
Neither good nor poor	92 (36.8)
Poor	12 (4.8)
Very poor	2 (0.8)
**The effect of COVID-19 disease on receiving the pregnancy care**	
Yes	76 (30.4)
No	174 (69.6)
**Prolonged spouse’s stay at home during the COVID-19 outbreak**	
Yes	151 (60.4)
No	99 (39.6)
**Reduction of the spouse income during the COVID-19 outbreak**	
Yes	193 (77.2)
No	57 (22.8)
**Aggressive behavior of the spouse during COVID-19 outbreak**	
Yes	39 (15.6)
No	211 (84.4)
**The effect of COVID-19 disease on the relationship with the spouse**	
Yes	54 (21.2)
No	196 (78.4)

^a^This variable was assessed subjectively and the participants’ responses were given by “very poor”, “poor” “moderate” and “rich”

More than one-third of participants (35.2 %) had experienced domestic violence during the COVID-19 outbreak. The most common type of violence experienced was emotional violence (32.8 %) followed by sexual (12.4 %) and physical violence (4.8 %). Additionally, 58 participants (23.2 %) in the violence-exposed group had experienced only one type of violence, 25 (10 %) two types, and five (2 %) three types. The main reported type of emotional violence was limiting the woman’s relationship with the family, friends, or neighbors (*n* = 46), yelling or insults (*n* = 40), and humiliation in the presence of others (*n* = 24). The main type of sexual violence was asking for sexual intercourse without the person’s consent (*n* = 21), use of compulsion and coercion for sexual intercourse (*n* = 11), and asking for atypical sexual actions without the woman’s consent (*n* = 11). Finally, the main type of physical violence included throwing objects (*n* = 7), pushing (*n* = 6), and pulling hair or arm (*n* = 5). None of the pregnant women reported threats with a knife, burning or scolding, or flogging. Remarkably, women experiencing moderate and severe violence reported more types of violence.

According to the violence categorization outlined above (1–2 times: mild violence, 3–5 times: moderate violence, and > 5: severe violence); the data indicated that 31 (37.8 %), five (16.2 %), and three (25 %) participants experienced respectively mild, moderate, and severe violence (Table 2). The results of chi-square and independent t-tests showed that domestic violence was significantly related to self-rated health status (*p* = 0.046), reduced spouse income during the COVID-19 pandemic (*p* = 0.017), aggressive behavior of the spouse during COVID-19 pandemic (*p* = 0.001), and the effect of COVID-19 disease on the relationship with the spouse (*p* = 0.001). However, domestic violence was not significantly related to other socio-demographic and obstetrics variables (*p* > 0.05).

**Table 2:** The frequency distribution of different types of domestic violence and their intensity among the pregnant women during the COVID-19 outbreak (n = 250)

Types of Violence	Total violence	Violence Mild	Moderate Violence	Severe Violence
	**Number (%)**	**Number (%)**	**Number (%)**	**Number (%)**
**Emotional violence**	82 (32.8)	29 (35.4)	22 (26.8)	31 (37.8)
**Sexual violence**	31 (12.4)	16 (51.6)	10 (32.2)	5 (16.2)
**Physical violence**	12 (4.8)	4 (33.3)	5 (41.7)	3 (25)

The total score of quality of life ranged between 0 and 100. Although the mean and standard deviation of quality of life in the physical dimension (total subscales of the physical health) were lower in the group of women exposed to violence (respectively 50.21 and 20.72) compared to the unexposed group (respectively 53.45 and 21.71), there was no significant difference between them (*p* = 0.25). However, the quality of life in the mental health department (total subscales of mental health) was significantly different between the said two groups (46.27 and 22.43 versus 61.17 and 21.28, respectively, with *p* < 0.001). In the exposed group, the highest mean score belonged to the general health subscale with a mean of 56.25; whereas the lowest mean score belonged to the emotional problems subscale, with a mean of 40.90. On the other hand, in the unexposed group of women, the highest mean score belonged to the emotional problems subscale, with a mean of 66.66, while the lowest mean score belonged to the physical functioning subscale, with a mean of 46.75. The results of the independent t-test indicated that quality of life was significantly different between the two groups of women in the following subscales: emotional problems (*p* < 0.001), social functioning (*P* = 0.002), psychological health (*p* < 0.001), and joy and energy (*p* = 0.009), (Table 3).

**Table 3:** Comparison of mean (SD) of quality of life in two groups of pregnant women with or without domestic violence

Quality of life	All (*n* = 250)	Violence (*n* = 88)	Non- Violence (*n* = 162)	*p*-value*
	Mean (SD)	Mean (SD)	Mean (SD)	
**Physical health**	52.31 (21.39)	50.21 (20.73)	53.45 (21.71)	0.25
General health	56.82 (19.53)	56.25 (20.50)	57.14 (19.04)	0.73
Bodily pain	58.30 (25.4)	54.26 (26.04)	60.49 (24.87)	0.064
physical health	48.00 (46.09)	44.31 (46.38)	50.00 (45.95)	0.35
Physical functioning	46.50 (27.44)	46.02 (27.56)	46.75 (27.46)	0.84
**Mental health**	55.93 (22.80)	61.17 (21.29)	46.28 (22.44)	< 0.001
Emotional Role	57.60 (49.51)	40.90 (49.44)	66.66 (47.28)	< 0.001
Social functioning	52.64 (27.48)	45.22 (27.70)	56.66 (26.58)	0.002
Mental health	59.96 (21.06)	50.79 (21.34)	64.93 (19.21)	< 0.001
Vitality	53.52 (23.73)	48.18 (25.12)	56.41 (22.50)	0.009

* Independent t-test

According to the independent t-test, this study found no relationship between violence and the quality of life score in the physical health department; thus, a general linear model was used to determine the relationship between violence and the quality of life score in the mental health department. The results indicated that while controlling the socio-demographic and obstetrics characteristics (economic status, the effect of COVID-19 disease on pregnancy care, having COVID-19, COVID-19 in relatives or acquaintances, prolonged spouse’s stay at home during the COVID-19 outbreak) as the possible confounding variables, the quality of life score was significantly higher in unexposed women compared to the women exposed to violence (β = 9.3, %95 CI = 3.5—15.0, p = 0.002), (Table 4).

**Table 4:** The relationship between domestic violence and mental health component of quality of life in pregnant women during the outbreak of COVID 19 in Iran based on the adjusted general linear model

Variable	Adjusted	
	**β (95 % Confidence Interval)**	***P*-value**
**Total violence (Reference: No)**	---	---
Yes	(15.03 3.51 to) 9.27	0.002
**Economic status (Reference: Very poor)**	---	---
Rich	(78.38 11.19 to-) 33.60	0.141
Moderate	(28.58 8.98 to-) 9.80	0.305
Poor	(25.79–12.63 to ) 6.58	0.501
**The effect of COVID-19 disease on receiving the pregnancy care (Reference:No)**	---	---
Yes	(3.53–8.17 to ) -2.32	0.436
**Prolonged spouse’s stay at home during the COVID-19 outbreak (Reference: No)**	---	---
Yes	(-1.02 -11.76 to ) -6.40	0.020
**Aggressive behavior of the spouse during COVID-19 outbreak (Reference: No)**	---	---
Yes	(2.68–15.55 to ) -6.43	0.166
**The effect of COVID-19 disease on the relationship with the spouse (Reference: No)**	---	---
Yes	(-1.72 -17.88 to ) -9.80	0.018

## Discussion

The results of the study indicated that more than one-third of participants were exposed to domestic violence. The most common type of violence experienced was emotional violence, followed by sexual and physical violence. The mean score of quality of life in the mental health department was significantly lower in the exposed women compared to their unexposed counterparts.

To the best of our knowledge, there is no study on the frequency of domestic violence in Iranian pregnant women during the COVID-19 pandemic; therefore, we compared our findings with those of studies before the COVID-19 pandemic. In the study by Hesami et al. [33], domestic violence during pregnancy was reported as 68.7 %. Tavoli et al. [27] also reported the occurrence of domestic violence against women during pregnancy in Iran, with 64.8 % of women exposed to mental or physical violence during their pregnancy, and similarly, Gharacheh et al. [34] reported that 44.5 % of women experience violence during pregnancy. Sarayloo et al. [35], reported a domestic violence rate of 46 %. In the research by Bahrami et al. [36], the prevalence of violence committed by a spouse was reported as about 67 %. Musa et al. [37] reported the prevalence of violence committed by a partner during pregnancy as 39.81 %. The prevalence of violence reported in this study was lower compared to the reviewed studies which could arise from ethnic differences, economic conditions, as well as the sociocultural context of the participants. Further, the reviewed studies focus on the underprivileged areas of Iran with different ethnicities; However, the current study was conducted in the city of Tabriz, which is one of the metropolises of Iran. The only study conducted in Tabriz was by Bahrami et al. [36], while the difference in the rate of domestic violence against pregnant women in the two studies could be due to differences among the participants and research tools. The mean age of pregnant women who participated in their study was 25.8; while the mean age of participants in this study was 30.6. A higher prevalence of domestic violence has been reported in younger women in other studies [38, 39]. A figure of 35 % domestic violence against pregnant women is still high, which can be considered a critical situation with potentially adverse effects on both maternal and neonatal health.

The main type of violence reported was emotional (32.8 %) followed by sexual (12.4 %) and physical (4.8 %) violence. Musa et al. [37] reported the prevalence of physical, emotional, and sexual violence as 25.93 %, 25.62 %, and 3.7 % respectively. The results of the study by Hessami et al. [33], Sarayloo et al. [35], and Tavoli et al. [27] were in line with our study, reporting higher levels of emotional- and lower levels of physical violence.

The main type of emotional violence reported in this study was limiting the woman’s relationship with her family, friends, or neighbors. In other studies conducted in Iran, this behavior has been attributed to attempting to isolate women and preventing the publicization of violence against them [27, 34, 35]. Nevertheless, remarkably the main cause of this behavior in this study was probably fearing that the pregnant mother and her fetus might contract the virus during the COVID-19 pandemic.

The findings of this study showed that domestic violence was significantly associated with the quality of life in pregnant women, with exposed women reporting lower quality of life. The women exposed to violence, compared to their unexposed peers, reported a lower quality of life concerning both mental and physical health, though the difference was significant only in the mental health department. Tavoli et al. [27] and Gharacheh et al. [34] reported that in Iran, domestic violence during pregnancy is negatively correlated with health-related quality of life. Behford et al. [40] reported that in China, the health-related quality of life was considerably lower in women experiencing sexual violence by their partner.

Our results indicated that four out of eight subscales of SF-12 (emotional problems, social functioning, psychological health, as well as joy and energy) were significantly lower in exposed women compared to their unexposed peers. A higher level of domestic violence meant a lower quality of life. Law et al. [24], who investigated the effect of violence committed by an intimate partner on the quality of life of women after delivery, found that the women who had experienced different types of violence committed by the intimate partner reported lower scores in most subscales of SF-36. Further, Gharacheh et al. [34] reported that six subscales of quality of life were significantly associated with domestic violence.

Pregnancy alone imposes a considerable psychological and physical strain on a person, and when accompanied by other stressful factors such as violence, it can have adverse effects on both neonatal and maternal health, with these complications causing increased maternal and neonatal mortality [1, 2, 41, 42]. During the COVID-19 pandemic, pregnant women bear more stress because of their special conditions. According to this study, they have a lower quality of life, and when domestic violence is added to these conditions, their situation becomes critical; therefore, maternal and fetal complications would increase [13, 14, 41].

The key finding is that this group of women need greater support during epidemics and disasters. In the course of the COVID-19 pandemic, the healthcare system is under great pressure, as COVID-19 has increased the load on hospitals and clinics; however, the health sector can take important measures to reduce the risk of violence against women during the pandemic, and help mitigate its adverse effects. Governments should develop the necessary plans to address violence against women during the COVID-19 pandemic. They should also provide the necessary human and financial resources and adopt the strategies to achieve them [22].

One of the limitations of the current study was its cross-sectional nature. As such, the relationship discovered between domestic violence and quality of life cannot be considered causal. Convenience sampling was another limitation, reducing the generalizability of the findings. The possibility of response bias is also important because of the nature of the research questions. By assuring the participants about confidentiality and anonymity, the authors attempted to minimize this limitation. Also, this study was performed in Tabriz city which has a Turkish ethnicity. As such, its results cannot be generalized to other cities and ethnicities of Iran. One of the main strengths of this study is being the first of its kind in Iran during the COVID-19 pandemic; therefore, it can offer essential information to healthcare experts and policymakers involved in women’s care.

## Conclusions

The results of this study indicated a high prevalence of domestic violence and its association with a lower quality of life among pregnant women during the COVID-19 pandemic. In Iran, pregnant women visit health centers during their pregnancy to receive healthcare services. Hence, it is vital to screen pregnant women in terms of domestic violence in these centers as well as other relevant centers, besides taking the proper interventions to address domestic violence and improve the quality of life in abused women. Thus, healthcare policymakers and experts involved in women’s care—particularly during dire, crisis conditions such as the COVID-19 pandemic—should be aware of the extent of this problem and take the proper measures to properly address the issue. Implementing effective solutions to prevent and address such violence against women is important, not only for women’s health, but also for the health of unborn children and other children in the family.

## Data Availability

Datasets used and analyzed during this study are available from the corresponding author on reasonable request.

## Abbreviations

WHO: World Health Organization
SF-12: Short Form Health Survey
COVID-19: Coronavirus disease 2019
95% CI: 95% Confidence Interval
SD: Standard Deviation
